# Acquisition of Host Cytosolic Protein by Toxoplasma gondii Bradyzoites

**DOI:** 10.1128/mSphere.00934-20

**Published:** 2021-01-27

**Authors:** Geetha Kannan, Pariyamon Thaprawat, Tracey L. Schultz, Vern B. Carruthers

**Affiliations:** a Department of Microbiology and Immunology, University of Michigan Medical School, Ann Arbor, Michigan, USA; b Medical Scientist Training Program, University of Michigan Medical School, Ann Arbor, Michigan, USA; Indiana University School of Medicine

**Keywords:** chronic infection, endocytosis, parasite, protease

## Abstract

Toxoplasma gondii is a protozoan parasite that persists in the central nervous system as intracellular chronic-stage bradyzoites that are encapsulated by a thick cyst wall. While the cyst wall separates bradyzoites from the host cytosol, it has been posited that small solutes can traverse the cyst wall to sustain bradyzoites. Recently, it was found that host cytosolic macromolecules can cross the parasitophorous vacuole and are ingested and digested by actively replicating acute-stage tachyzoites. However, the extent to which bradyzoites have an active ingestion pathway remained unknown. To interrogate this, we modified previously published protocols that look at tachyzoite acquisition and digestion of host proteins by measuring parasite accumulation of a host-expressed reporter protein after impairment of an endolysosomal protease (cathepsin protease L [CPL]). Using two cystogenic parasite strains (ME49 and Pru), we demonstrate that T. gondii bradyzoites can ingest host-derived cytosolic mCherry. Bradyzoites acquire host mCherry within 4 h of invasion and after cyst wall formation. This study provides direct evidence that host macromolecules can be internalized by T. gondii bradyzoites across the cyst wall in infected cells.

**IMPORTANCE** Chronic infection of humans with Toxoplasma gondii is common, but little is known about how this intracellular parasite obtains the resources that it needs to persist indefinitely inside neurons and muscle cells. Here, we provide evidence that the chronic-stage form of T. gondii can internalize proteins from the cytosol of infected cells despite residing within an intracellular cyst that is surrounded by a cyst wall. We also show that accumulation of host-derived protein within the chronic-stage parasites is enhanced by disruption of a parasite protease, suggesting that such protein is normally degraded to generate peptides and amino acids. Taken together, our findings imply that chronic-stage T. gondii can ingest and digest host proteins, potentially to support its persistence.

## Abstract (precis)

Chronic infection of humans with Toxoplasma gondii is common, but little is known about how this intracellular parasite obtains the resources that it needs to persist indefinitely inside neurons and muscle cells. Here, we provide evidence that the chronic-stage form of T. gondii can internalize proteins from the cytosol of infected cells despite residing within an intracellular cyst that is surrounded by a cyst wall.

## INTRODUCTION

Toxoplasma gondii is a global pathogen that impacts numerous mammalian species. Infection with this parasite can lead to blindness and encephalitis in humans as a result of reactivated infection (1, 2). The underlying source of these diseases is the chronic form of the parasite, characterized by slowly replicating bradyzoite cysts. Under specific circumstances, bradyzoites can reconvert to actively replicating tachyzoites, which induces inflammation and contributes to disease progression (3). While therapeutics exist to effectively combat tachyzoites, there is currently no fully effective treatment for bradyzoites (4). A better understanding of how bradyzoites persist is necessary to inform rational therapeutic targeting of this elusive stage of T. gondii.

There is a growing cognizance of the heterogeneous and dynamic nature of bradyzoite cysts (5). During developmental switching from tachyzoite to bradyzoite, parasite proteins are secreted and localized adjacent to the parasitophorous vacuolar membrane (PVM) to form the thick glycan-rich cyst wall (6). As the cyst matures, the composition and localization of T. gondii proteins in the wall are altered while bradyzoites continue to replicate (7, 8). These processes require a continued source of nutrients, yet it is unclear from where bradyzoites acquire such resources and what they are.

One possibility is that host macromolecular proteins help satisfy the energy and anabolic requirements of bradyzoite cysts. The cyst wall acts as a barrier between bradyzoites and the host cytoplasm, akin to the PVM separating tachyzoites from the host cytoplasm. Yet tachyzoites can acquire across the PVM small solutes (dyes of ∼1 kDa; presumably amino acids and nucleotides, etc.) and macromolecules (green fluorescent protein [GFP] and mCherry) from the host cytoplasm (9–12). It has been proposed that tachyzoites ingest host cytoplasmic proteins via endocytosis at the micropore (13, 14). Micropores have also been observed in bradyzoite cysts (13). In addition, dyes (<10 kDa) and horseradish peroxidase (HRP) (44 kDa) taken up by host cells via endocytosis have been found to enter the cyst matrix and bradyzoites within *in vitro* cysts, whereas larger proteins such as bovine serum albumin (BSA) (65 kDa) and transferrin (80 kDa) were observed only around the cyst wall (11, 15). Although these studies on bradyzoites suggest that solutes and proteins of certain sizes that are endocytosed by host cells can be acquired by the chronic stage of T. gondii, they do not directly address the extent to which bradyzoites acquire proteins derived from the cytosol of infected cells.

To determine whether bradyzoites ingest host cytosolic protein, we adapted previously published protocols that demonstrated the uptake, trafficking, and accumulation of host cytosolic mCherry in tachyzoites (9, 10, 16, 17). Here, we validate experimental conditions to be used to interrogate bradyzoite ingestion (e.g., parasite conversion conditions and doxycycline [DOX] treatment) on tachyzoites and provide evidence of host-derived cytosolic mCherry uptake by T. gondii bradyzoites.

## RESULTS

### Parasite conversion conditions and doxycycline treatment.

Tachyzoites have previously been shown to ingest host cytosolic mCherry from transiently transfected and doxycycline-induced Chinese hamster ovary (CHO) cell lines (16). A buildup of host-derived fluorescent protein within tachyzoites is clearly visible when parasite digestion is impaired by either genetic ablation or chemical inhibition of cathepsin protease L (CPL) (9, 10, 16, 17). Ingested material is observed under standard CHO cell culture conditions (5% CO_2_, 10% serum [pH 7.1]) and without parasite exposure to doxycycline. However, *in vitro*, bradyzoites are generated under conversion-inducing conditions (ambient CO_2_, 5% serum [pH 8.3]) and would be exposed to doxycycline during the induction of host mCherry. Doxycycline has been reported to have anti-*Toxoplasma* activity *in vitro* and *in vivo* (18) and thus may impact parasite ingestion. Therefore, we first sought to test whether host cytosolic mCherry can be detected within tachyzoites that ingest under conversion-inducing conditions and after exposure to doxycycline.

**FIG 1:**
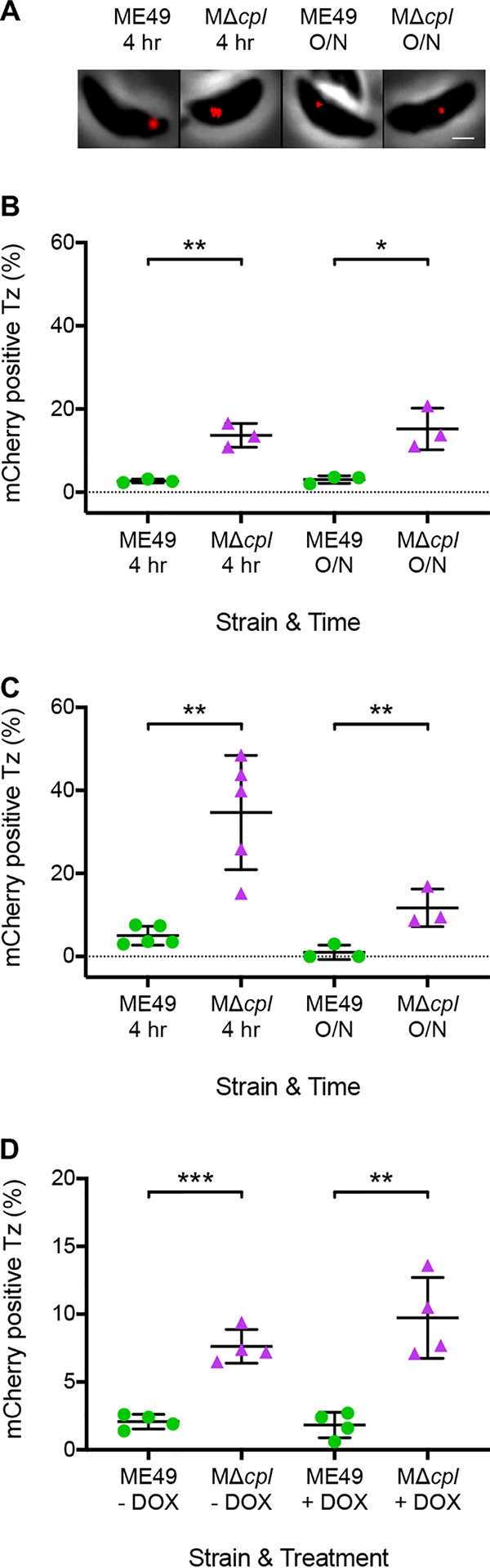
Ingestion of host cytosolic material by tachyzoites under bradyzoite-inducing conditions and doxycycline treatment. (A) Representative images of tachyzoites with ingested host cytosolic mCherry under bradyzoite-inducing conditions. Bar, 1 μm. (B) Ingestion of host cytosolic mCherry by T. gondii tachyzoites (Tz) under bradyzoite-inducing conditions. Three independent experiments were performed for each strain. The following parasites were enumerated for each experiment: ME49 for 4 h (280, 265, and 256 parasites), MΔ*cpl* (304, 271, and 303), ME49 overnight (O/N) (199, 228, and 220), and MΔ*cpl* overnight (236, 195, and 287). An unpaired *t* test was performed to compare genotypes within each time point. * denotes a *P* value of <0.05, and ** denotes a *P* value of <0.005. (C) Ingestion of host cytosolic mCherry by T. gondii tachyzoites under CHO cell culture conditions. Three or five independent experiments were performed. The following parasites were enumerated for each experiment: ME49 for 4 h (281, 227, 259, 244, and 262 parasites), MΔ*cpl* (163, 228, 263, 283, and 272), ME49 overnight (194, 180, 220, 340, 293, and 529), and MΔ*cpl* overnight (218, 295, 231, 328, 238, and 322). A Mann-Whitney U test was performed to compare genotypes within each time point. * denotes a *P* value of <0.05, and ** denotes a *P* value of <0.005. (D) Ingestion of host cytosolic mCherry by T. gondii tachyzoites after doxycycline treatment. Four independent experiments were performed. The following parasites were enumerated for each experiment: ME49 −DOX (318, 271, 258, and 210 parasites), MΔ*cpl* −DOX (214, 277, 230, and 292), ME49 +DOX (226, 338, 248, and 206), and MΔ*cpl* +DOX (324, 235, 222, and 266). An unpaired *t* test was performed to compare strains within each treatment. ** denotes a *P* value of <0.005, and *** denotes a *P* value of <0.0005.

To assess the extent to which parasite conversion conditions and doxycycline treatment might impair the ability of parasites to acquire host cytosolic mCherry, we performed tachyzoite ingestion assays on wild-type (WT) ME49 and genetically ablated CPL (MΔ*cpl*) tachyzoites (Fig. 1). Prior to tachyzoite infection, mCherry expression was induced in inducible CHO (iCHO) cells for 4 to 5 days with doxycycline. Undigested mCherry was observed within tachyzoites ingesting under conversion-inducing conditions (Fig. 1A). We found a significant increase in the number of mCherry-positive MΔ*cpl* tachyzoites compared to the WT under conversion-inducing conditions (Fig. 1B). This increase was observed in MΔ*cpl* at 4 h postinfection and after overnight replication of parasites in mCherry-expressing iCHO cells. The same results were observed under standard CHO cell culture conditions, which served as a positive control for the assay (Fig. 1C). In addition, there was an increase in the number of mCherry-positive MΔ*cpl* compared to WT tachyzoites that were pretreated with doxycycline (+DOX) for 5 days, which was also observed in untreated (−DOX) controls (Fig. 1D). Taken together, these findings indicate that host cytosolic mCherry can be ingested by tachyzoites under conversion-inducing culture conditions and with exposure to doxycycline.

### Bradyzoite ingestion of host-derived mCherry.

**FIG 2:**
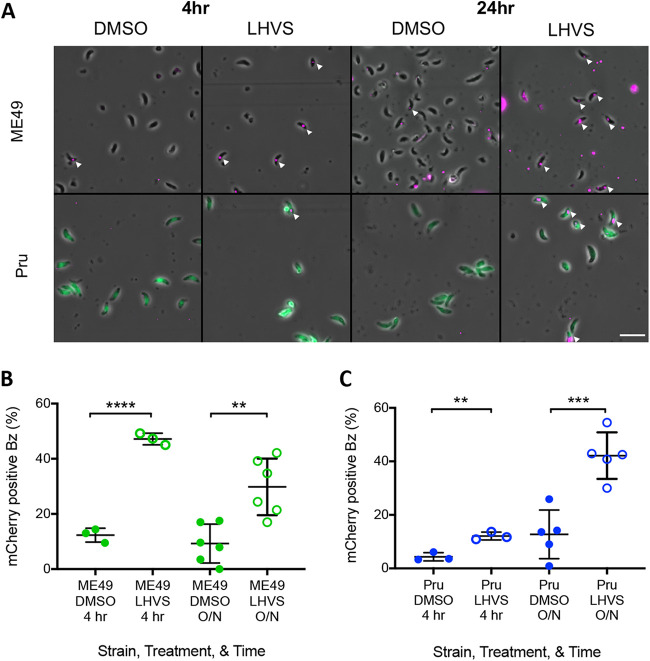
Bradyzoites can ingest host-derived mCherry. (A) Representative fields of view of bradyzoites with ingested host cytosolic mCherry. Bar, 10 μm. White arrowheads point to bradyzoites containing host-derived mCherry. (B) Bradyzoites (Bz) from mouse brain cysts can ingest host cytosolic mCherry. Three to six independent experiments were performed. The following parasites were enumerated for each experiment: ME49 with DMSO for 4 h (236, 210, and 277 parasites), ME49 with LHVS for 4 h (10 μM) (255, 299, and 245), ME49 with DMSO overnight (O/N) (282, 229, 217, 245, 228, and 245), and ME49 with LHVS (3 μM) overnight (266, 394, 234, 245, 202, and 217). An unpaired *t* test was performed to compare treatments within each time point. ** denotes a *P* value of <0.005, and **** denotes a *P* value of <0.0001. (C) Ingestion of host cytosolic mCherry by *in vitro* bradyzoites. Three to five independent experiments were performed. The following GFP^+^ parasites were enumerated for each experiment: Pru with DMSO for 4 h (365, 231, and 214 parasites), Pru with LHVS for 4 h (1 μM) (213, 238, and 175), Pru with DMSO overnight (231, 189, 154, 212, and 231), and Pru with LHVS (1 μM) overnight (318, 245, 213, 200, and 242). An unpaired *t* test was performed to compare treatments within each time point. ** denotes a *P* value of <0.005, and *** denotes a *P* value of <0.0005.

We next wanted to determine whether bradyzoites can ingest host cytosolic mCherry. To do this, we performed the ingestion assay with either *in vivo-* or *in vitro*-derived bradyzoites that were liberated from cysts (Fig. 2). Because an insufficient number of MΔ*cpl* cysts can be recovered from the rodent brain (19), we used the irreversible CPL inhibitor morpholinurea-leucine-homophenylalanine-vinyl phenyl sulfone (LHVS) to limit parasite digestion during and after invasion. Host-derived mCherry was observed in bradyzoites under all conditions (Fig. 2A). As with MΔ*cpl* tachyzoites (Fig. 1A), a significantly higher percentage of LHVS-treated *in vivo-*derived ME49 bradyzoites contained host mCherry than the dimethyl sulfoxide (DMSO) control (Fig. 2B). This was seen both at 4 h postinfection and after overnight replication. We found the same results with *in vitro-*derived bradyzoites of a separate cystogenic strain, Pru (Fig. 2C). Taken together, these data suggest that T. gondii bradyzoites can acquire host cytosolic mCherry within 24 h of invasion.

### Ingestion of mCherry after cyst wall formation.

**FIG 3:**
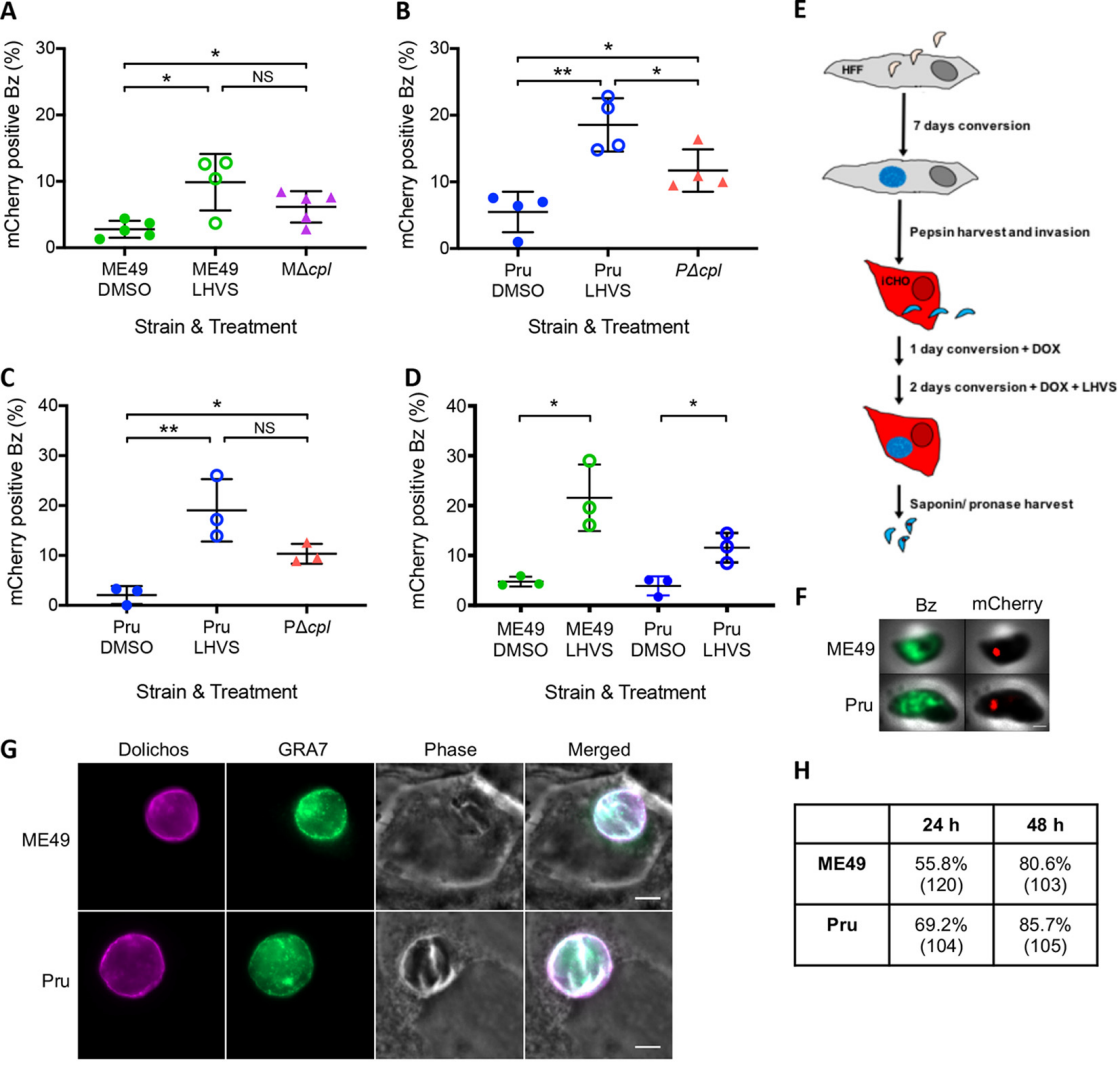
*In vitro* bradyzoite cysts contain host-derived mCherry. (A) Ingestion of host cytosolic mCherry by T. gondii *in vitro* bradyzoites (Bz) converted in iCHO cells for 12 days and treated with LHVS for 2 days. BAG1^+^ stained parasites were enumerated. Four to five independent experiments were performed for each strain and treatment. The following parasites were enumerated for each experiment: ME49 with DMSO (262, 342, 272, 237, and 272 parasites), ME49 with LHVS (231, 195, 328, and 231), and MΔ*cpl* (95, 254, 282, 135, and 251). A Mann-Whitney U test was used to compare groups. * denotes a *P* value of <0.05, and ** denotes a *P* value of <0.005. NS, not significant. (B) Ingestion of host cytosolic mCherry by T. gondii *in vitro* bradyzoites converted in iCHO cells for 12 days and treated with LHVS for 2 days. GFP^+^ parasites were enumerated. Four independent experiments were performed for each strain and treatment. The following parasites were enumerated for each experiment: Pru with DMSO (85, 277, 264, and 298 parasites), Pru with LHVS (209, 215, 223, and 317), and PΔ*cpl* (270, 432, 232, and 276). An unpaired *t* test was used to compare all groups. * denotes a *P* value of <0.05, and ** denotes a *P* value of <0.005. (C) Ingestion of host cytosolic mCherry by T. gondii *in vitro* bradyzoites converted in iCHO cells for 8 days and treated with LHVS for 2 days. GFP^+^ parasites were enumerated. Three independent experiments were performed for each strain and treatment. The following parasites were enumerated for each experiment: Pru with DMSO (270, 210, and 209 parasites), Pru with LHVS (357, 227, and 209), and PΔ*cpl* (464, 198, and 224). An unpaired *t* test was used to compare all groups. * denotes a *P* value of <0.05, and ** denotes a *P* value of <0.005. (D) Ingestion of host cytosolic mCherry by T. gondii *in vitro* bradyzoites converted in HFFs for 7 days, harvested, and then converted in iCHO cells for 3 days with 1 μM LHVS for 2 days. BAG1^+^ ME49 and GFP^+^ Pru bradyzoites were enumerated. Three independent experiments were performed for each strain and treatment. The following parasites were enumerated for each experiment: ME49 with DMSO (234, 97, and 205 parasites), ME49 with LHVS (281, 112, and 220), Pru with DMSO (230, 144, and 156), and Pru with LHVS (212, 186, and 209). (E) Schematic of the experimental design for panel D. (F) Representative LHVS-treated bradyzoites from panel D. The bradyzoite marker is BAG1 for ME49 and LDH2 promoter-driven expression of GFP for Pru. Bar, 1 μm. (G) Representative images of intracellular ME49 and Pru cysts at 24 h postinfection. Bars, 5 μm. (H) Percentage of dolichos-positive vacuoles at 24 and 48 h postinfection. The total numbers of GRA7 vacuoles counted are listed in parentheses. Data are from one biological replicate.

We next wanted to determine whether bradyzoites can acquire host-derived mCherry through the cyst wall (Fig. 3). To elucidate this, we infected iCHO cells with ME49 or Pru tachyzoites and converted them to bradyzoites for 7 days prior to doxycycline induction for 5 days. There was a significant increase in host-derived mCherry upon chemical and genetic ablation of CPL in ME49 (Fig. 3A) and Pru (Fig. 3B) bradyzoites compared to WT DMSO controls. The same results were obtained when Pru parasites were converted for a shorter time (Fig. 3C). However, when in culture for nearly a week, *in vitro* bradyzoites can egress from a cyst to invade adjacent host cells. Because the bradyzoites in these experiments were in culture for 8 to 12 days, it is possible that the ingested mCherry that we observe is from newly invading parasites rather than parasites passing through the cyst wall. To address this, we shortened the time of conversion in iCHO cells to 3 days by starting with purified *in vitro*-derived bradyzoites, and we washed off extracellular bradyzoites after a 4-h invasion. After 1 day of infection under bradyzoite differentiation conditions, we added LHVS and continued treatment for 2 days. Under these conditions, we again found a higher percentage of LHVS-treated ME49 and Pru parasites with mCherry than in DMSO controls (Fig. 3D). A schematic for this experiment is provided in Fig. 3E, and representative images of bradyzoites with ingested mCherry are shown in Fig. 3F. We stained infected monolayers to assess the formation of the cyst wall (Fig. 3G) and found that more than 50% of parasitophorous vacuoles (GRA7 positive [GRA7^+^]) were positive for the cyst wall marker dolichos at day 1 postinfection, while >80% stained for the cyst wall at 2 days postinfection (Fig. 3H). Taken together, these data suggest that T. gondii bradyzoites can acquire host cytosolic protein through the cyst wall.

## DISCUSSION

Here, we provide evidence that *Toxoplasma* bradyzoites can ingest host cytosolic mCherry. This acquisition of host protein by bradyzoites occurs within 4 h of invasion as well as after 24 h of bradyzoite infection, when the cyst wall would have started forming (7). Our findings are therefore consistent with host proteins potentially serving as a nutrition source for bradyzoite cysts.

Our study builds upon prior discoveries that the cyst wall allows the passage of large dyes (<10 kDa) and HRP (44 kDa) that can be incorporated into bradyzoites (11, 15). The utilization of tracers of different sizes for bulk and receptor-mediated endocytosis revealed that bradyzoites may incorporate only proteins of up to 44 kDa via bulk endocytosis (15). However, these tracers are taken up by the host cell from the extracellular environment through endocytosis and trafficked to the host lysosomes that decorate the outside of the cyst wall, and material up to a certain size is then taken into the cyst. It is uncertain whether the host-endocytosed material enters bradyzoites via bulk or receptor-mediated endocytosis. Nevertheless, in contrast to these studies, ours shows another source of resources for bradyzoites: host-derived cytosolic proteins.

There are two limitations to our study. The first consideration is that host cytosolic mCherry within bradyzoites may have been acquired in tachyzoite-like parasites (e.g., not fully mature bradyzoites). Indeed, it has been shown in studies that tachyzoites are capable of ingesting host cytosolic mCherry (10, 16, 17). Although tachyzoites are killed during the pepsin harvesting of bradyzoites from cysts, we cannot rule out the possibility that partially converted tachyzoites within *in vitro* cysts survived harvesting and were the ones to ingest host material or that once added to the culture, fully mature bradyzoites from the mouse brain did not begin expressing tachyzoite proteins that would aid in ingestion. Live-cell imaging with parasites expressing stage-specific fluorescent proteins and host cells that express cytoplasmic proteins that fluoresce only once inside the parasite lysosome would aid in clearly demonstrating that bradyzoites are capable of ingesting host cytosolic protein.

The second limitation is that we do not know the maturity of *in vitro* cysts within iCHO cells. It has recently been shown that the architecture and composition of the cyst wall change during maturation (7, 8). While we found host-derived mCherry within bradyzoites that had CPL inhibited after the cyst wall had started forming, it is conceivable that the wall mirrored that of an immature cyst. It is therefore unclear whether host-derived proteins would be capable of passing through a fully mature cyst wall, as would be found *in vivo.* Staining and analysis of the localization of different proteins within the cyst wall could discern the maturity of *in vitro* cysts and at what level of development the parasites may stop utilizing host cytosolic proteins for nutrients and instead move to an alternative energy source.

In order for T. gondii to survive and thrive, the parasite acquires a multitude of nutrients from the host cell via various mechanisms. For instance, tachyzoites obtain host-derived fatty acids, proteins, and small molecules/amino acids to support replication. While host-derived protein can be acquired within minutes of parasite invasion and after the PVM has formed, small molecules and amino acids are obtained after PVM formation through the GRA17/23 putative nutrient pore, and fatty acids traverse the PVM during tachyzoite replication (9, 12, 20). It would be unsurprising that with their newly appreciated dynamic qualities, bradyzoite cysts also persist by obtaining nutrients from the host cell through different sources. For example, *in vitro*, T. gondii bradyzoites form more lipid droplets when oleic acid (OA) is added to the culture media, suggesting parasite acquisition of host-derived fatty acids (21). More recently, GRA17 has also been posited to provide nutrients to bradyzoite cysts during differentiation, suggested by the decreased viability of ME49 Δ*gra17* parasites (22). Our current study adds host cytoplasmic proteins to the list of host-derived components that bradyzoites can acquire.

The mechanism, source, and rate by which T. gondii acquires nutrients are likely influenced by metabolic requirements during the life stage and health of the parasite. It is possible that a genetic loss or chemical inhibition of CPL attenuates the recycling of parasite proteins and results in greater uptake of host proteins. Indeed, the high percentage of mCherry-positive tachyzoites in Fig. 1C compared to the percentages under other conditions or with bradyzoites might be attributed to a greater nutrient requirement for tachyzoites than for bradyzoites. The lower numbers of mCherry-positive tachyzoites that invade under conversion conditions could indicate shifting of metabolic requirements as the parasites are exposed to stressors to induce conversion to bradyzoites.

To better understand the importance of host cytosolic protein acquisition by T. gondii bradyzoites for parasite persistence, more experiments are needed to gain clarity and mechanistic insight. As mentioned above for the second caveat to this study, it would be important to have an understanding as to the point in cyst maturation that host-derived proteins are a key source of nutrients compared to the other sources. In addition, it is necessary to elucidate a mechanism by which T. gondii bradyzoites take up host cytoplasmic protein. Inhibiting the acquisition of host-derived protein uptake would enable studies to determine the importance of this nutrient pathway in the viability and persistence of T. gondii bradyzoites.

## MATERIALS AND METHODS

### Parasite cultures.

The following strains were used in this study: ME49 wild type (WT), ME49 deficient in CPL (MΔ*cpl*), Pru Δ*ku80*SLUC (Pru), and Pru Δ*ku80*SLUC deficient in CPL (PΔ*cpl*). Pru Δ*ku80SLUC* expresses GFP under control of the early bradyzoite promoter LDH2. Details on the generation of these strains were previously described (9, 19). T. gondii tachyzoites were maintained in human foreskin fibroblast (HFF) monolayers grown under standard conditions (16).

### mCherry ingestion assays.

Parasite ingestion was determined using modifications of a previously reported assay (16). In brief, mCherry was expressed in inducible Chinese hamster ovary (iCHO) cells with the addition of 2 μg/ml of doxycycline (DOX). Parasites from infected iCHO cells were harvested, purified, treated with pronase and saponin, and imaged on Cell-Tak (Fisher Scientific)-coated slides using a Zeiss Axiovert Observer Z1 inverted fluorescence microscope. For each biological replicate, more than 97 parasites of each genotype or treatment were enumerated for host-derived mCherry accumulation within parasites. Samples were coded during the time of harvesting to blind the experimenter during imaging and quantification.

In this study, we refer to the *in vitro* conditions that promote tachyzoite conversion to bradyzoite cysts as “bradyzoite-inducing conditions.” This includes the use of alkaline (conversion) media and growth without CO_2_. For all experiments, 2 μg/ml DOX was used. Detailed changes for each ingestion assay are described below.

### (i) Tachyzoite ingestion under CHO versus conversion-inducing conditions.

mCherry was induced with DOX for 4 days in iCHO cells grown in standard CHO growth medium (nutrient rich) (10% cosmic calf serum, penicillin-streptomycin [Pen/Strep] [pH 7.2]) or conversion medium (RPMI 1640 without NaHCO_3_, 50 mM HEPES, 5% cosmic calf serum, Pen/Strep [pH 8.3]). To assess ingestion after overnight infection, iCHO cells were infected with tachyzoites after 4 days of induction and kept under growth and induction conditions during infection. To assess ingestion after 4 h of infection, iCHO cells were infected after 5 days of induction and kept under growth conditions during infection. Tachyzoites were then harvested and enumerated as described above.

### (ii) Tachyzoite ingestion with doxycycline treatment.

The effect of doxycycline on ingestion was assessed in the following manner. WT (i.e., not expressing mCherry) CHO cells were infected with tachyzoites and grown under standard growth conditions for 5 days with DOX. In parallel, uninfected iCHO cells were grown under parasite conversion conditions with DOX to induce mCherry. After 5 days, tachyzoites were harvested from WT CHO cells and allowed to invade iCHO cells under conversion conditions for 4 h while being kept under growth conditions. Tachyzoites were then harvested and enumerated as described above.

### (iii) Ingestion by purified *in vitro*-derived bradyzoites.

Tachyzoites were converted to bradyzoite cysts in HFFs under HFF-specific conversion conditions (RPMI 1640 without NaHCO_3_, 50 mM HEPES, 3% fetal bovine serum [FBS], penicillin-streptomycin [pH 8.2]) for 7 days. During the last 2 days, parasites were treated with either 1 μM morpholinurea-leucine-homophenylalanine-vinyl phenyl sulfone (LHVS) or dimethyl sulfoxide (DMSO) as the solvent control. After this time, bradyzoites were harvested from *in vitro* cysts using pepsin treatment (16). Purified bradyzoites were allowed to invade iCHO cells under conversion conditions in the presence of DOX, LHVS (1 μM), and DMSO either for 4 h or overnight. Parasites were then harvested using saponin/pronase, and the number of mCherry^+^ parasites of GFP^+^ parasites was enumerated.

### (iv) Ingestion of purified *in vivo*-derived bradyzoites.

Eight-week-old male CBA/J mice (Jackson Laboratories) were infected with ME49 WT tachyzoites and humanely sacrificed at 5 weeks postinfection according to protocols approved by the University of Michigan’s Animal Care and Use Committee. Brains from infected mice were homogenized in 1 ml of sterile Hanks’ buffered salt solution (HBSS), and bradyzoites were subsequently harvested using pepsin treatment (23). Purified bradyzoites invaded iCHO cells under conversion conditions in the presence of DOX, LHVS, and DMSO either for 4 h (10 μM LHVS) or overnight (3 μM LHVS). Parasites were then harvested using saponin/pronase, and the number of mCherry^+^ parasites was enumerated.

### (v) Ingestion by *in vitro* bradyzoite cysts.

Assessment of ingestion by *in vitro* bradyzoites within the cyst was done in two ways. In one set of experiments, tachyzoites were converted to bradyzoite cysts in iCHO cells under conversion conditions for 8 to 12 days. During the last 5 days of conversion, mCherry was induced with DOX. Parasites were treated with 1 μM LHVS or DMSO for the last 2 days of induction. In a second set of experiments, tachyzoites were converted to bradyzoite cysts within HFFs for 7 days. Bradyzoites were harvested via pepsin treatment and allowed to invade iCHO cells for 4 h before washing with medium to remove extracellular bradyzoites. Bradyzoites were then kept under conversion conditions for 3 days, with the addition of DOX and 1 μM LHVS or DMSO for the last 2 days. iCHO cells had been treated with DOX for 2 days prior to bradyzoite infection. At the end of each experiment, bradyzoites were harvested from *in vitro* cysts with saponin/pronase. The number of mCherry^+^ parasites of GFP^+^ or bradyzoite antigen 1-positive (BAG1^+^) parasites was enumerated.

### Bradyzoite staining.

Purified bradyzoites were fixed in 4% paraformaldehyde, permeabilized in 0.5% Triton X-100, and stained for BAG1 with primary rabbit anti-TgBAG1 (1:400) (generated by immunization of rabbits with Escherichia coli-derived recombinant BAG1) and secondary goat anti-rabbit 488 (1:1,000) (Invitrogen).

### Cyst wall formation.

To measure the extent to which the cyst wall had formed around bradyzoites at 24 h postinfection, we performed the experiment described above in the second series of experiments for ingestion by *in vitro* bradyzoite cysts. Rather than performing the bradyzoite harvest with saponin/pronase, infected iCHO cells were fixed in 4% formaldehyde 24 and 48 h after bradyzoite infection. Fixed cells were permeabilized with 0.1% Triton X-100 for 10 min, blocked with blocking buffer (10% FBS–0.01% Triton X-100 in phosphate-buffered saline [PBS]), and stained with primary biotinylated dolichos (1:400) (Vector Laboratories) and mouse anti-GRA7 (clone 12B6) (1:1,000) (Peter Bradley) and secondary streptavidin Alexa Fluor 350 (1:1,000) (Thermo Fisher Scientific/Invitrogen) and goat anti-mouse Alexa Fluor 488 (1:1,000) (Thermo Fisher Scientific/Invitrogen). Images were taken on a Zeiss Axio Observer Z1 inverted microscope at a ×63 magnification and analyzed using Zen blue edition software. The numbers of GRA7^+^ vacuoles and GRA7^+^ and dolichos-positive cysts were enumerated to calculate the percentage of cysts.

### Statistics.

Data were analyzed using GraphPad Prism. For each data set, outliers were identified and removed using ROUT with a *Q* value of 0.1%. Data were then tested for normality and equal variance. If the data passed both tests, unpaired Student’s *t* test or one-way analysis of variance (ANOVA) with Dunn’s multiple-comparison test was performed. If the data failed one or both tests, a Mann-Whitney U test or a Kruskal-Wallis test was performed.
